# Laser-assisted endoscopic removal of a foreign body impacted in the esophagus

**DOI:** 10.1016/j.igie.2025.01.004

**Published:** 2025-01-17

**Authors:** Rohit Gupta, Sugata Narayan Biswas

**Affiliations:** 1 Foundation for Research & Education in Gastroenterology & Endoscopy, Prayagraj, Uttar Pradesh, India; 2 Department of Gastroenterology, Moti Lal Nehru Medical College, Prayagraj, Uttar Pradesh, India

A 72-year-old man presented with dysphagia and chest pain after accidental ingestion of a denture a week earlier. Computed tomography showed a foreign body impacted in the upper esophagus without any evidence of perforation. On upper endoscopy, a denture (Fig. 1A and B) was found snugly fit below the upper esophageal sphincter (Video 1, available online at www.igiejournal.org). Initially, using a forceps, we attempted to grasp the denture base and disimpact the flanges from the esophageal wall. However, because of a wide denture base and flanges firmly impacted on the esophageal wall, it was difficult to dislodge. Holmium laser-assisted endoscopic removal of the denture was planned and discussed with the patient. The denture base was fragmented using holmium laser (10 W for 2 J at 5 Hz) (Fig. 1C and D). Subsequently, 1 limb of a grasping forceps was inserted in the central hole of the denture base created by laser disintegration, and the denture base was firmly gripped (Fig. 1E). Thereafter, 1 of the flanges was disimpacted from the esophageal wall and admitted into an overtube, and the entire denture was carefully extracted (Fig. 1F and G). A relook upper endoscopy showed deep ulcers of the esophageal wall at the site of denture impaction (Fig. 1H). The patient was kept fasted for 2 days and was discharged without any adverse events.

**Figure 1:**
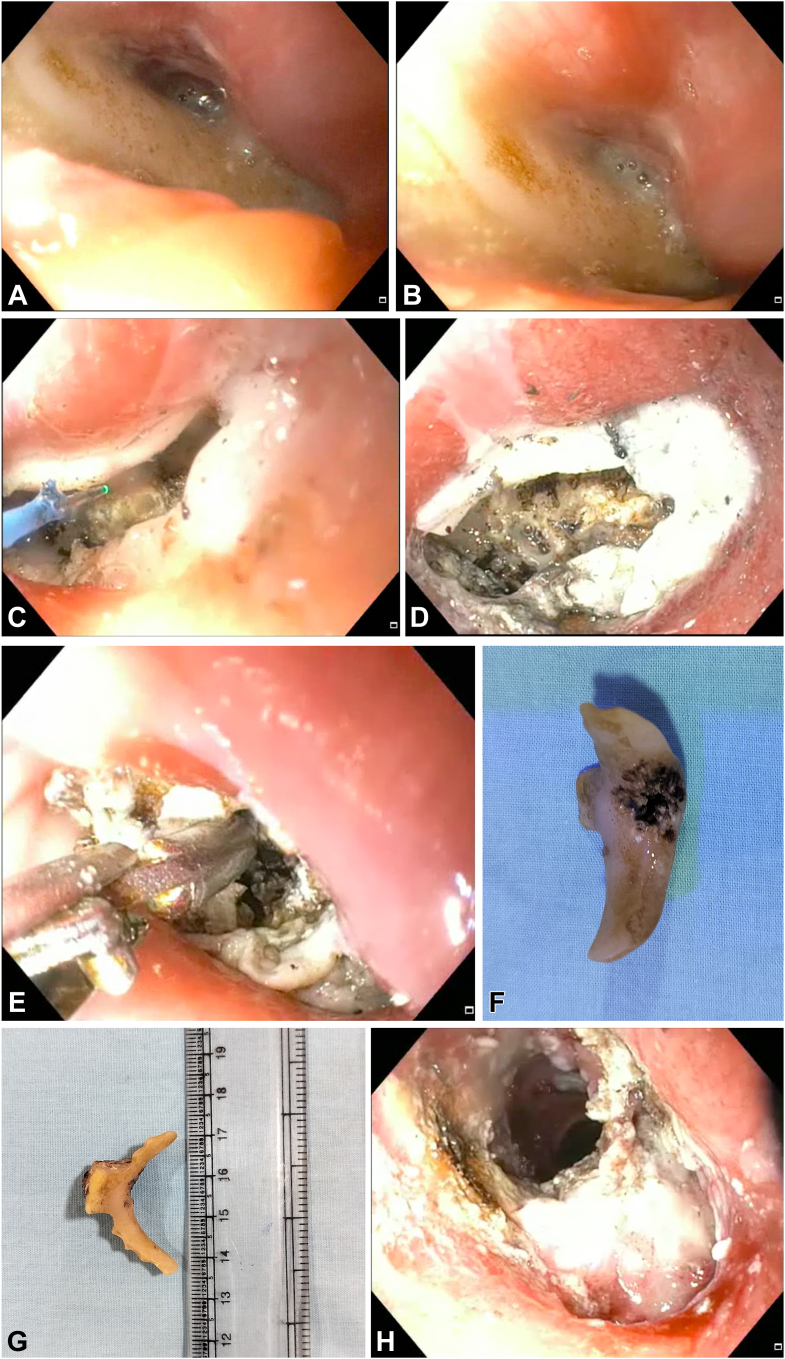
**A** and **B,** Upper endoscopy showing the denture impacted below the upper esophageal sphincter. **C** and **D,** The denture base disintegrated by the holmium laser. **E,** Grasping forceps used to extract the denture after laser fragmentation. **F** and **G,** The denture after extraction. **H,** Esophageal ulcer at the site of denture impaction.

Foreign bodies impacted in the esophagus are often encountered in clinical practice and bear the risk of esophageal perforation. Endoscopic removal using forceps, snares, biliary baskets, and balloons usually aids in the safe removal of foreign bodies. When conventional techniques fail, laser-assisted fragmentation and disimpaction may facilitate extraction of a foreign body impacted in the gastrointestinal tract. Laser-assisted removal of foreign bodies from the stomach and colon has been previously reported in the literature.^1^, ^2^, ^3^, ^4^, ^5^ However, the use of laser-assisted fragmentation as an effective modality in the removal of impacted esophageal foreign bodies has rarely been reported.^6^^,^^7^ This case demonstrates a novel technique for safe removal of difficult and impacted esophageal foreign bodies.

## Patient consent

The patient in this article has given written informed consent to publication of his case details.

## Disclosure

All authors disclosed no financial relationships.

## Supplementary Material

Video 1 Video showing holmium laser–assisted removal of a denture impacted in the esophagus.
